# T-Cell-Rich Angiomatoid Polypoid Pseudolymphoma: A Case Report and Comparison With Key Differential Diagnoses

**DOI:** 10.7759/cureus.37241

**Published:** 2023-04-07

**Authors:** Katie R Xu, Bethany R Rohr

**Affiliations:** 1 Dermatology, Case Western Reserve University School of Medicine, Cleveland, USA; 2 Dermatology, University Hospitals Cleveland Medical Center, Cleveland, USA

**Keywords:** lymphoproliferative disoder, pseudolymphoma, vascular tumor, immunohistochemistry, t-cell-rich angiomatoid polypoid pseudolymphoma

## Abstract

T-cell-rich angiomatoid polypoid pseudolymphoma (TRAPP) is a rare and recently defined entity, conceptualized just over a decade ago. Recognition of TRAPP is important because it can be clinically and microscopically confused with low-grade cutaneous lymphomas and other vascular proliferations. We report a case of a 28-year-old male with a solitary 1.2 cm red polypoid papule on the middle posterior base of the neck. The histopathological examination revealed a well-circumscribed dermal nodular proliferation of banal-appearing lymphovascular spaces with plump endothelial cells. Immunohistochemical analysis showed a T-cell-rich infiltrate. The clinical-pathological differential diagnosis for TRAPP includes pyogenic granuloma, angiolymphoid hyperplasia (epithelioid hemangioma), acral pseudolymphomatous angiokeratoma of children, cutaneous lymphoid hyperplasia, and low-grade cutaneous lymphomas and lymphoproliferative disorders. We review the literature and discuss the key differentiating features between TRAPP and its common differential diagnoses.

## Introduction

T-cell-rich angiomatoid polypoid pseudolymphoma (TRAPP) is a rare cutaneous vascular proliferation that presents as a solitary polypoid papule mainly on the head and neck of adults, with a slight female predominance [1,2]. In contrast to low-grade lymphomas or other cutaneous vascular neoplasms, most literature characterizes TRAPP as a solitary, exophytic erythematous papule with a striking dermal-based T-cell-rich lymphocytic infiltrate and prominent vessels lined by plump endothelial cells [1,3]. Herein, we report an additional case of TRAPP with a review of the literature and comparison to entities in the clinical and histopathological differential diagnoses.

## Case presentation

A 28-year-old male with no known relevant past medical history presented to his family physician for evaluation of a 1.2 cm red polypoid plaque on the middle posterior base of the neck. Shave excision was completed, and the specimen was submitted to dermatopathology with the differential diagnosis of hemangioma.

**Figure 1:**
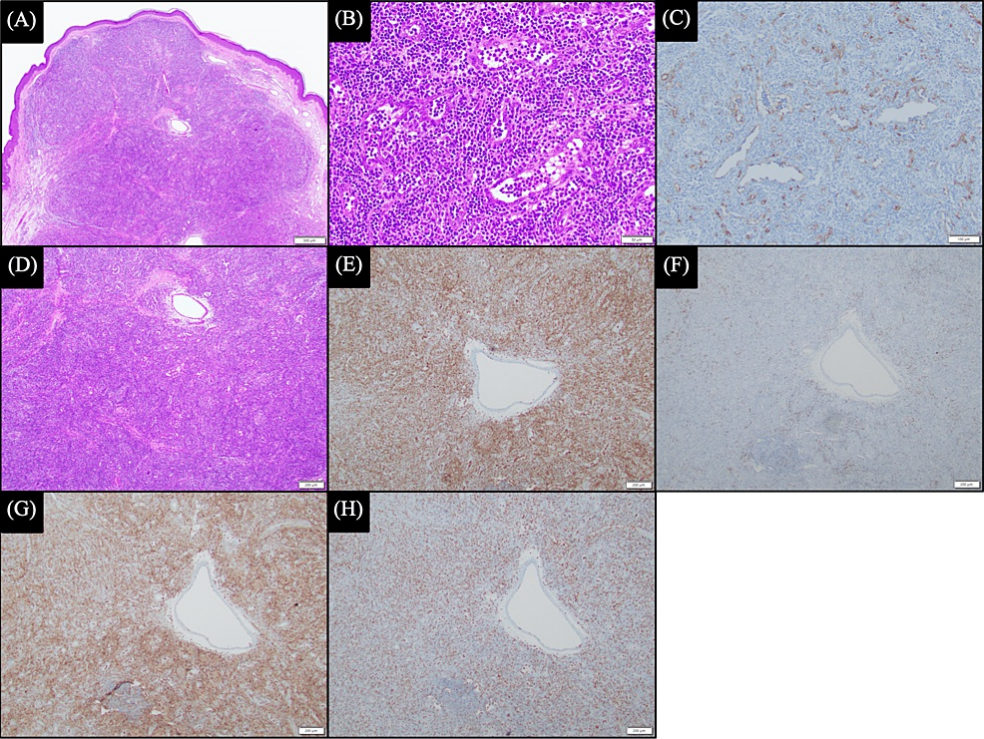
T-cell-rich angiomatoid polypoid pseudolymphoma. (A) Scanning magnification reveals a dome-shaped profile with dermal infiltrate (H&E, x20). (B) High magnification reveals banal-appearing lymphocytic infiltrate surrounding vascular spaces with plump endothelial cells (H&E, x200). (C) Plump CD34+ endothelial cells (x100). (D) High magnification reveals a mononuclear cell infiltrate and increased vascular spaces (H&E, x40). (E) CD3+ T-cell infiltrate (x400). (F) Background population of CD20+ B-cells (x400). (G) CD4 immunostain (x400). (H) CD8 immunostain (x400).

The specimen revealed an exophytic, dome-shaped profile with a well-circumscribed dermal nodular proliferation of banal-appearing lymphovascular spaces lined by plump CD34+ endothelial cells (Figure 1A-C) with a dense surrounding mononuclear cell infiltrate (Figure 1D). The majority of these small cells were CD3+ T-cells with a lesser background population of CD20+ B-cells (Figure 1E and F). The CD4+ to CD8+ ratio was approximately 2:1 (Figure 1G and H). A diagnosis of TRAPP was rendered. The patient was followed by his family physician, and clinical course records were not available.

## Discussion

TRAPP is characterized by a solitary, polypoid, exophytic erythematous papule that most commonly arises on the head and neck [1]. TRAPP typically occurs in young adults [2], with a slight female predominance [1]. Its pathogenesis is unknown [2]. TRAPP does not have a tendency for local recurrence [2], and no recurrences after lesional excision have been reported in one study of 17 cases with a follow-up range from 24 to 120 months [1].

Histopathology shows a polypoid profile with prominent lymphovascular spaces lined by plump endothelial cells. There is a dense lymphocytic infiltrate separated from the epidermis by a grenz zone [2,4]. The infiltrate is composed of CD3+ T-cells with a mix of CD4+ and CD8+ cells [1,2]. The proportion of CD4+ to CD8+ T-cells have been inconsistently reported in the literature, with some reports suggesting CD4+ predominance [1] and others suggesting CD8+ predominance [3]. Scattered B-cells, eosinophils, and plasma cells may be seen [2]. Mild lymphocytic atypia has been reported [4].

Evolving proposals of nomenclature changes contribute to the conceptual ambiguity and diagnostic difficulty of TRAPP and similar lymphoid vascular tumors. Various terms have been proposed over the past several decades to describe this spectrum of lesions with similar morphological and immunophenotypic findings, including papular angiolymphoid hyperplasia [5], papular angiolymphoid proliferation with epithelioid features in adults and children (PALE-FACE) [6], and angiolymphoid hyperplasia with high endothelial venules (ALH-HEV) [7]. The appellation, TRAPP, was proposed just over a decade ago [1]. More recently, the term ‘inflammatory lobular hemangioma (ILH)’ was proposed to describe the spectrum of related cutaneous vascular lesions including TRAPP, APACHE, PALE-FACE, and ALH-HEV [8]. ‘ILH’ was proposed in place of ‘pseudolymphoma’ to emphasize the primary classification of these lesions as vascular proliferations and neoplasms, accompanied by a dense lymphocytic inflammatory infiltrate [8]. For the purposes of this discussion, the term ‘TRAPP’ will be used to describe the entity in question.

TRAPP has significant clinical and histopathological resemblance with other vascular neoplasms including pyogenic granuloma (PG), angiolymphoid hyperplasia with eosinophilia (ALHE) (also known as epithelioid hemangioma), Kimura’s disease (eosinophilic lymphogranuloma), cutaneous epithelioid angiomatous nodule (CEAN), and acral pseudolymphomatous angiokeratoma of children (APACHE). The dense lymphocytic infiltrate in TRAPP may be mistaken for low-grade cutaneous lymphomas, lymphoproliferative disorders, and pseudolymphomas including primary cutaneous marginal zone B-cell lymphoproliferative disorder (PCMZLD), primary cutaneous follicle center lymphoma (PCFCL), primary cutaneous CD4+ small/medium pleomorphic T-cell lymphoproliferative disorder (PCSM-TCLPD), and cutaneous lymphoid hyperplasia (CLH). Comparisons between these entities are summarized in Table 1.

**Table 1:** Differential diagnosis of TRAPP. TRAPP: T-cell-rich angiomatoid polypoid pseudolymphoma; ALHE: angiolymphoid hyperplasia with eosinophilia; IFN-α: interferon alpha; HHV: human herpesvirus; CMV: cytomegalovirus; CEAN: Cutaneous epithelioid angiomatous nodule; EBER: Epstein-Barr encoding region; APACHE: acral pseudolymphomatous angiokeratoma of children; PCMZLD: primary cutaneous marginal zone B-cell lymphoproliferative disorder; PCFCL: primary cutaneous follicle center B-cell lymphoma; PCSM-TCLPD: primary cutaneous CD4+ small/medium T-cell lymphoproliferative disorder; PD-1: programmed cell death protein 1; CLH: cutaneous lymphoid hyperplasia; BCL: B-cell lymphoma; TCR: T-cell receptor.

Entity	Demographic	Clinical	Histopathology	Immunohistochemistry	Treatment	Prognosis
TRAPP	Mostly adults; female predominance [1,2]	Single polypoid erythematous papule [1,2]; 2.5-7.5 mm diameter [1]; common sites: head, neck [1]	Epidermal collarette, grenz zone [2,4]; prominent vessels lined by plump endothelial cells [1,2]; T-cell-rich lymphocytic dermal infiltrate [1,2]; admixed plasma cells and histiocytes [4]	Admixture of CD4+ and CD8+ T-cells [1,2]; vessels stain with CD31 and CD34	Surgical excision [1]	Benign [1]; no tendency for recurrence [1,2]
Pyogenic granuloma (PG)	Mostly children and young adults [1,2]; female predominance in childbearing years [9]	Usually single pedunculated or sessile red, ulcerated, friable, papule with collarette [9]; common sites: gingiva, lips, mucosa of the nose, face, fingers [9,10]	Epidermal collarette [9]; lobular capillary proliferation separated by fibrous areas [2,9]; degree of inflammatory infiltrate depends on ulceration; usually a mix of neutrophils and lymphocytes [2]	GLUT-1- [9]; vessels stain with CD31 and CD34	Surgical excision; electrodesiccation; topical treatments: corticosteroids, β-blockers [10]	Benign [1]; multiple recurrences are more common in adolescents and young adults, typically following electrodesiccation or surgical removal; lesions may resolve spontaneously within a few months or following withdrawal of causative drug or parturition [10]
ALHE	Mostly young to middle-aged adults [1,2,9]	Single or multiple [1] pink to red-brown painless [2] papules or nodules [9]; common sites: face, scalp, ears, neck [2,9]	Vascular proliferation with plump endothelial cells and surrounding lymphoid aggregates with eosinophils [2,3,9]; surrounding proliferation of smaller vessels [9]	Vessels stain with CD31 and CD34; HHV-8- [2]	Surgical excision; laser therapy (pulsed dye laser); cryosurgery; electrosurgery; topical or intralesional steroids; IFN-α; bleomycin; tacrolimus ointment; anti-IL-5 antibody mepolizumab [9]	Benign [2,11]; common recurrence following excision [1,2]
Kimura’s disease (eosinophilic hyperplastic lymphogranuloma)	Mostly young to middle-aged adults; Asian, male predominance [2,9,12]	Single or multiple subcutaneous skin-colored tender [2] nodules or tumors [12]; typically 2-5 cm diameter [12]; common sites: periauricular, parotid glands, neck [9,12]; elevated serum immunoglobulin E and peripheral blood eosinophilia [2,9,12]; 50-60% of patients have regional lymphadenopathy [9]	Reactive lymphoid follicles with germinal centers with background eosinophils, plasma cells, and mast cells [2,9,12]; sometimes eosinophilic microabscesses [2,9,12]; vascular hyperplasia, hyalinization, and fibrosis [12]; thin, flat endothelial cells [9,12]	Clonal populations of T-cells in some patients [9]; vessels stain with CD31 and CD34	Surgical excision; regional or systemic corticosteroid therapy; cyclosporine; radiotherapy [9]	Benign [12]; recurrence is common (up to 60-80%) [12]; death is rare [9,12]
CEAN	Mostly adults [2,13]	Single or multiple non-encapsulated, well-circumscribed, solitary erythematous papules or nodules; common sites: trunk and extremities [2,13]	Sheets of large epithelioid endothelial cells [1] with conspicuous nucleoli and abundant eosinophilic to clear cytoplasm [2,9,13]; infiltrate of lymphocytes, plasma cells, and occasional eosinophils [13]; mild-to-moderate cytological atypia of endothelial cells [13]; background fibrosis and hemosiderin deposition [2,9]	Variably CD31+ and CD34+; HHV-8- and CMV-; negative Warthin-Starry stain and EBER in situ hybridization [13]	Surgical excision; cryotherapy [9]	Benign [1,9,13]; excision is curative [9,13]
APACHE	Mostly children and adolescents [1-3,9]; female predominance [8]	Single or multiple [1] reddish brown papules or plaques, often in a linear cluster [2,9,11]; common sites: acral [1-3,9]	Upper dermal lymphoid hyperplasia [1,9]; proliferation of small vessels lined by plump endothelial cells [2,9]; infiltrate composed of small lymphocytes, histiocytes, plasma cells, eosinophils, and occasional giant cells [9]	Admixture of CD4+ and CD8+ T-cells [2,9]	Surgical excision or curettage; topical or intralesional steroids; cryotherapy; radiotherapy [14]	Benign [1,14]; least recurrence with surgical excision [14]
PCMZLD	Mostly middle-aged adults [2,15]; male predominance [9,15]	Single or multiple red or violet papules, nodules, and plaques; common sites: arms and trunk [2,9]	Patchy, nodular, or diffuse dermal lymphoid infiltrate composed of small lymphocytes, marginal zone B-cells, and plasma cells [9,15]; reactive germinal centers surrounded by paler zones [9]	CD20+, CD79a+, BCL-2+, CD5-, CD10-, CD23-, cyclin D1-, monotypic plasma cells [2,9]; clonal rearrangement of IgH [9]	Radiotherapy; surgical excision; chemotherapy; rituximab; IFN-ɑ [15]	50% recurrence; systemic dissemination and death are uncommon; 5-year survival rate >95% [15]
PCFCL	Mostly middle-aged adults [16] male predominance [16]	Usually single, red or violet plaque or tumor; common sites: head, neck, and trunk [2,9]	Nodular or diffuse dermal infiltrate with atypical germinal centers [2,9]	CD20+, CD79a+, and BCL-6+ [16]; CD5-, cyclin D1- [2]; clonal rearrangement of IgH in >50% of cases [9]	Radiotherapy; surgical excision; rituximab; IFN-ɑ [17]	Local radiation therapy: 99% complete response rate [16]; 30% relapse rate [16]; 5-year survival rate >95% [2,9]
PCSM-TCLPD	Mostly adults [2]	Single or multiple pink or violet papules, plaques, and tumors [2]; common sites: head and neck [2,18]	Nodular or diffuse dermal infiltrate of small- to medium-sized lymphocytes; can have neutrophils, eosinophils, and plasma cells [2]	CD3+, CD4+, CD8-, CD30- [18]; monoclonal TCR rearrangements [2]; grouped PD-1 expression in follicular helper T-cells [18]	Surgical excision; radiotherapy; intralesional corticosteroids; chemotherapy [18]	High cure rates from excision and radiotherapy [18]
CLH	Mostly young adults [1,11]; female predominance [1,9]	Single or multiple red-brown or violet papules or nodules [9,11]; common sites: head and neck [1,8,11]	Nodular or diffuse dermal infiltrate with lymphoid follicles and reactive germinal centers [9,11]; admixed histiocytes, plasma cells, and eosinophils [9]	Predominance of T-cells, B-cells, or a near equal mixture of both; reactive germinal centers: BCL-6+, CD10+, BCL-2- [11]	Corticosteroids; surgical excision; radiotherapy; immunosuppressants [11]	Benign [1,11]; lesions may resolve spontaneously after months or years [9]; treatment of underlying disease or instigating agent can be curative [11]

Pyogenic granuloma

TRAPP is most often clinically misdiagnosed as pyogenic granuloma [1]. Both PG and TRAPP have been observed in a wide range of ages, from adolescents to older adults [1,2]. While PG often presents on the head like TRAPP, its common sites also include the mucous membranes of the gingiva, lips, and nasal mucosa [9,10], which are not observed in TRAPP. PG is usually ulcerated [9], unlike TRAPP. Histologically, the lack of a well-developed lobular capillary proliferation and the presence of a brisk lymphocytic infiltrate in TRAPP draw a distinction from PG [1].

Angiolymphoid hyperplasia with eosinophilia

Like TRAPP, ALHE often presents on the head and neck regions of adults [1,9]. However, ALHE presents as grouped papules [1] rather than single lesions. In contrast to TRAPP, ALHE is characterized by plump epithelioid endothelial cells protruding into vascular lumens in a “hobnail” fashion [3,19]. TRAPP lacks this multilobular vascular pattern with a feeder vessel [1,3]. ALHE features a diffuse inflammatory infiltrate dominated by lymphoid aggregates with eosinophils [3,9,20], which is not observed in TRAPP. Additionally, TRAPP is not known to recur, while ALHE often recurs [1,2]. Fos proto-oncogene (FOS) and FosB proto-oncogene (FOSB) immunohistochemical expression are associated with ALHE [2,20], more frequently than TRAPP. In one report, FOSB and FOS were expressed in four and three of 11 TRAPP cases, respectively, while FOSB and FOS co-expression were observed in seven of nine ALHE cases [3].

Kimura’s disease

 Unlike TRAPP, Kimura’s disease can present as grouped papules as well as solitary lesions, with common recurrence (22% recurrence described in one study of 41 patients) [12] and predominance in Asian men [2,9,12]. Additionally, TRAPP lacks the lymphoid follicles, eosinophilic infiltrate, and regional lymphadenopathy characteristic of Kimura's disease [2,9,12].

Cutaneous epithelioid angiomatous nodule

Like TRAPP, CEAN is a cutaneous vascular proliferation that may present as a well-circumscribed solitary erythematous papule [1,2,13]. CEAN commonly occurs on the trunk and extremities [2,13], which are uncommon sites for TRAPP. While both feature a lymphocytic infiltrate, CEAN is composed of sheets of large epithelioid cells that are not observed in TRAPP [1,2,9,13].

Acral pseudolymphomatous angiokeratoma of children

 TRAPP shares nearly identical histopathologic features of APACHE. The main distinguishing feature is the clinical setting. TRAPP presents as a solitary polypoid papule on non-acral sites, mainly in adults, whereas APACHE presents as multiple acral papules or plaques on the hands and feet, mainly in children [1-3,9], although some cases of APACHE have been reported in middle-aged and elderly adults [14]. Additionally, TRAPP features a T-cell-rich infiltrate, while APACHE features a more heterogeneous infiltrate, featuring small lymphocytes, histiocytes, plasma cells, eosinophils, and occasional giant cells [9].

Low-grade cutaneous lymphomas and lymphoproliferative disorders

On histopathology, TRAPP may raise diagnostic concerns for several lymphoproliferative disorders. Compared to PCMZLD and PCFCL, TRAPP is T-cell rich and lacks germinal centers or expanded marginal zones [1]. The predominance of T-cells in TRAPP may raise concern for cutaneous T-cell lymphomas. However, TRAPP lacks significant lymphocyte atypia and epidermotropism seen in mycosis fungoides [2,9]. Monoclonal T-cell receptor (TCR) rearrangement should not be seen in TRAPP. PCSM-TCLPD has small- to medium-sized pleomorphic CD4+/CD8- lymphocytes and PD1+ cells in clusters [18], which are not expected in TRAPP. The prominent vascular component in TRAPP is not a feature of cutaneous lymphomas or lymphoproliferative disorders.

Cutaneous lymphoid hyperplasia

Like TRAPP, CLH occurs on the upper extremities, trunk, head, and neck of adults [11] with a female predominance [1,9], but CLH does not classically appear as a polypoid papule. TRAPP lacks germinal center formation, which is often seen in CLH [9,11]. Additionally, the prominent vascular pattern [1] seen in TRAPP would be unusual for CLH.

## Conclusions

TRAPP clinically presents as a solitary raised red papule or nodule on the head and neck of adults. Histopathologically, TRAPP reveals a well-circumscribed vascular proliferation with plump endothelial cells and a dense T-cell-rich lymphocytic infiltrate. This case report and literature review highlights the clinical and histopathologic features to distinguish TRAPP from lymphomas, lymphoproliferative disorders, pseudolymphomas, and other vascular proliferations and neoplasms.
